# Evaluation of reservoirs in bleaching trays for at-home bleaching: a split-mouth single-blind randomized controlled equivalence trial

**DOI:** 10.1590/1678-7757-2020-0332

**Published:** 2020-08-17

**Authors:** Eveline Claudia MARTINI, Michael Willian FAVORETO, Fabiana Madalozzo COPPLA, Alessandro Dourado LOGUERCIO, Alessandra REIS

**Affiliations:** 1 Universidade Estadual de Ponta Grossa Departamento de Dentística Restauradora Ponta GrossaParaná Brasil Universidade Estadual de Ponta Grossa , Departamento de Dentística Restauradora , Ponta Grossa , Paraná , Brasil .; 2 Universidade Paranaense Departamento de Dentística Restauradora Francisco BeltrãoParaná Brasil Universidade Paranaense , Departamento de Dentística Restauradora , Francisco Beltrão , Paraná , Brasil .; 3 Centro de Ensino Superior dos Campos Gerais Departamento de Dentística Restauradora Ponta GrossaParaná Brasil Centro de Ensino Superior dos Campos Gerais (CESCAGE), Departamento de Dentística Restauradora , Ponta Grossa , Paraná , Brasil .

**Keywords:** Tooth bleaching, Dentin sensitivity, Clinical trial, Reservoirs, Carbamide peroxide

## Abstract

**Objectives:**

This randomized, split-mouth, single-blinded trial assessed whether the use of reservoirs in at-home bleaching trays is equivalent to non-reservoir trays. Our choice of an equivalence trial was based on the expectation that a non-reservoir tray is sufficient to produce a color change. Secondary outcomes such as tooth sensitivity (TS) and gingival irritation (GI) were also assessed.

**Methodology:**

Forty-six patients were selected with canines shade A2 or darker. In half of the patient’s arch, bleaching trays were made with reservoirs and the other half, without reservoirs. At-home bleaching was performed with carbamide peroxide (CP) 10% (3 h daily; 21 days). Color change was evaluated with a digital spectrophotometer (ΔE, ΔE00, and Whiteness Index) and shade guide units (ΔSGU) at baseline, during and one-month post-bleaching. TS and GI were assessed with a numeric scale (NRS) and a visual analog scale (VAS).

**Results:**

After one month, the equivalence of reservoir and non-reservoir groups were observed in all color instruments (p>0.05). Fifteen and sixteen patients presented pain (absolute risk: 33% and 35%, 95%, confidence interval (CI) 21-46% and 23-49%) in the reservoir and non-reservoir side, respectively. The odds ratio for pain was 0.8 (95%CI 0.2-3.0) and the p-value was non-significant (p=1.0). TS intensity was similar between both groups in any of the pain scales (p>0.05). No difference in the GI was observed (p>0.05).

**Conclusions:**

The protocol with reservoirs is equivalent in color change to the non-reservoir, although no superiority of the latter was observed in terms of reduced TS and GI with at-home 10% carbamide peroxide bleaching.

**Clinical Relevance:**

The presence of reservoirs in a bleaching tray did not improve color change or affect tooth sensitivity and gingival irritation.

## Introduction

Dental bleaching is widely used to make teeth whiter and brighter, a common desire among patients. ^1 - 3^ The dentist-supervised dental bleaching technique can be performed using high-concentrate materials (in-office protocol) or by dispensing low concentrate-material in a custom bleaching tray (at-home use).

Among these available protocols, clinicians consider at-home bleaching safer as it employs low concentrate products ^2^ and therefore reduces the risk and intensity of tooth sensitivity. ^4 , 5^ Additionally, it is an easy protocol, requires reduced chair-time, and it is cheaper than the in-office protocol.

Since the introduction of at-home bleaching, several modifications of the protocol and materials occurred in the past years. Carbamide peroxide or hydrogen peroxide with varied concentrations can now be employed. ^6^ The daily usage time of the bleaching tray was reduced ^7 , 8^ and modifications in the manufacture of the bleaching trays ^9^ were proposed with the presence of reservoirs. ^2 , 10 , 11^

Reservoirs are modifications in the tray molds to increase the amount of bleaching material carried by the bleaching tray, seeking greater bleaching efficacy. Fisher first introduced the use of tray reservoirs in 1992. ^12^ For such purpose, light-cured block-out resin or light-curing composites are applied on the buccal surface of teeth from the cast models to create an additional space between the tray and the teeth. The first report on the efficacy of reservoirs in bleaching trays come from the end of the 1990s. ^11^ This as well as other clinical studies ^11 , 13 , 14^ contested the efficacy of tray reservoirs in bleaching trays.

In a recent systematic review, the authors concluded that the majority of the studies that compared non-reservoir and reservoir were at unclear risk of bias, indicating the need for well-designed clinical trials. ^15^ Some important aspects of well-designed clinical studies such as randomization, allocation concealment, and blinding were missing in the eligible studies ^10 , 11 , 13 , 14 , 16 - 18^ and the studies lacked standardized methods for reporting important outcomes, such as color change, tooth sensitivity, and gingival irritation. This prevented the authors from this systematic review ^15^ to conclude on the study’s findings but to ask for the conduction of additional clinical trials that answer the same research question, since this modification related to the bleaching protocol, despite having the first reports published a long time ago, still generates controversies about its real effectiveness.

Therefore, the objective of this study was to conduct a randomized controlled equivalence trial with a split-mouth design to test that non-reservoir trays are ‘as effective as’ reservoir trays in terms of color change. The secondary outcomes risk of tooth sensitivity, intensity of tooth sensitivity, and gingival irritation were compared in a traditional superiority hypothesis testing.

## Methodology

After approval by the Ethics Committee for the Protection of Human subjects of the local university (protocol number 2.124.508), we registered the research protocol in the Brazilian Clinical Trials Registry (REBEC) under the identification number RBR-4w9ht3.

For the report of this clinical trial, we followed the recommendations of the CONSORT statement (Consolidated Standards of Reporting Trials statement) with extension for noninferiority and equivalence trials and within-person designs. ^19 , 20^ The explanatory CONSORT document can be found at the website www.consort.org.

### Study setting and locations

This was a randomized, split-mouth, and single-blind controlled equivalence trial. The clinical phase of the current study was performed from April 5, 2018, to October 15, 2018, in the Clinics of the School of Dentistry from the Universidade Estadual de Ponta Grossa.

### Recruitment and eligibility criteria

Patients for this clinical trial were recruited through social media. Volunteers that met the eligibility criteria read and signed an informed consent form before being enrolled in the study. To facilitate communication between the research staff and the volunteers, we set up a social network group via WhatsApp ^®^ .

Volunteers were required to be between 18 to 40 years old and in good general health (self-reported by the patient as not being under medical treatment) and good oral health (not in need of surgical, endodontic, periodontal and restorative treatment). The participants were required to have maxillary anterior teeth without caries, restorations and/or endodontic treatment. Canines needed to be shade A2 or darker (Vita Classical shade guide, Vita-Zahnfabrik, Bad Säckingen, Germany).

Patients who had already undergone tooth bleaching, using orthodontic apparatus, prosthesis, with severe internal tooth discoloration (tetracycline stain, fluorosis or endodontic treatment) were excluded from the study. Additionally, pregnant and lactating women, patients with bruxism or pathologies that could cause some type of sensitivity (gingival recession, dentin exposure, visible cracks) and patients taking anti-inflammatory or analgesic drugs were not included in the study.

### Sample size calculation

We designed this study to demonstrate equivalence in color change between at-home bleaching with reservoirs and without reservoirs. The sample size calculation showed that a minimal of 30 participants (alpha of 5%; power 90%) would be necessary to demonstrate an equivalence of 3 units of ∆E. The standard deviation of the ∆E after 3-week bleaching with a 10% carbamide peroxide was reported to be around 3.5 units, ^21^ and this value was used for sample size calculation. Due to the high number of volunteers for bleaching, a total of 46 participants took part in this controlled trial. The equivalence margin was previously specified based on earlier studies that reported that only ∆E values higher than 3.3 are clinically perceptible. ^22^

### Randomization and allocation concealment

A randomized list was generated by computer (www.sealedenvelope.com) and the allocation sequence was inserted into opaque sealed envelopes numbered from 1 to 46. The patients were numbered according to the sequence of enrollment. These envelopes were opened by the operator only at the time of the intervention. The treatment in the upper right arch was determined by the information contained in the envelope, while the other arch received alternative treatment.

### Blinding

This study was a randomized, single-blind controlled trial, in which the evaluator was blinded to the group assignment. A researcher not involved in the implementation and evaluation process was responsible for the delivery and guidance on the administration of the bleaching trays.

### Intervention

Two dentists, with more than 5 years of clinical experience (E.M. and F.M.C.) performed the bleaching procedure. They made alginate impressions of each participant’s jaw, and after disinfection, filled them with dental stone. The upper arch models were used in the study. In one of the sides, a photopolymerized blocking material (TopDam, FGM, Joinville, SC, Brazil) was applied in the buccal surfaces of the central, lateral, canine and premolar teeth in one side of the patient’s mouth to create reservoirs on these teeth.

The blocking resin was applied so that the labial surface was covered except for 1 mm in the mesial, distal and cervical axes. The other half arch had no reservoirs. The randomization processed defined the side that would receive the reservoirs. A 1.0 mm soft vinyl material (Ultradent Products, South Jordan, UT, USA) was used to fabricate the custom-fitted trays that would hold the whitening gel (Plastivac P7, BioArt, São Carlos, Brazil). The excess material from the labial and lingual surfaces was trimmed to 1 mm away from the gingival margin.

We instructed all participants to use the bleaching tray with the bleaching agent (10% carbamide peroxide with potassium nitrate and fluoride, Opalescence PF, Ultradent Products) for 3 hours once a day for 21 days. They were instructed to place an amount of gel to cover the buccal surface of all teeth (this amount being slightly higher in the reservoir-side of the bleaching tray). Participants were instructed to remove the tray after each bleaching period, rinse teeth with water and brush their teeth as usual.

As a measure of adherence to the experimental protocol study, participants received a diary in which they were asked to take note of the number of hours a day they used the tray during treatment. They were reminded of this procedure daily using the social media network group set up at the beginning of the study protocol.

### Outcomes

#### Color evaluation

For the evaluation of this primary outcome, two experienced and calibrated dentists (kappa statistics higher than 80% after previous calibration), who were not involved in the randomization procedures, performed clinical assessments at baseline, after each week of bleaching and one month after the bleaching treatment.

We performed the calibration procedure using 20 volunteers. The operators color-checked the canines independently, using shade guides, and when differences were noted, they had to reach an agreement. This procedure was repeated until they get a kappa equal to or higher than 80% in two consecutive measurements.

We performed the color evaluation using the shade guide VITA Classical and the VITA Bleachedguide 3D-MASTER. We also performed an objective color evaluation with the spectrophotometer VITA Easyshade (VITA Zahnfabrik, Bad Säckingen, Germany).

We arranged the Vita Classical scale in 16 tabs from highest (B1) to lowest (C4) value: B1, A1, B2, D2, A2, C1, C2, D4, A3, D3, B3, A3.5, B4, C3, A4, C4. The VITA Bleachedguide 3D-MASTER contains lighter shade tabs, and it is already organized from highest (0M1) to lowest (5M3) value. The selected tooth matching area was the middle third of the buccal surface of the upper canines. Color changes were calculated from the beginning of the active phase up to the specific recall times by calculating the change in the number of shade guide units (∆SGU), which occurred toward the lighter end of the value-oriented list of shade tabs. In case of disagreement between operators, they were required to reach consensus.

We created a jig made of dense silicone with a central window of 6 mm of radius to fit with the spectrophotometer tip and allow for standardization of color measurement. With the jig into position in the canines, the tip of the spectrophotomer was then inserted into the silicone guide to obtain the L*, a*, and b* parameters of color at the different time assessments. The L* value represents the luminosity (value from 0 [black] to 100 [white]), a* value represents the measurement along the red-green axis, and b* value represents the measurement along the yellow-blue axis. The color change (∆E) before (baseline) and after each treatment (in each assessment period) was given by differences between the two colors measured with the spectrophotometer, which was calculated using the CIELab formula from 1976: ∆E = [(∆L*) ^2^ + (∆a*) ^2^ + (∆b*) ^2^ ] ^1^ . Additionally, the color changes were calculated using the CIEDE 2000 formula: ^23^ ∆E00 = [(ΔL/kLSL) ^2^ + (ΔC/kCSC) ^2^ + (ΔH/kHSH) ^2^ + RT(ΔC*ΔH/SC*SH)] ^1^ and Whiteness Index proposed by Gerlach, Zhou and McClanahan ^24^ (2002): ∆Wi = [(100 – L _i_ *) ^2^ + a _i_ * ^2^ + b _i_ * ^2^ ] ^1^ .

#### Tooth sensitivity evaluation

We instructed patients to fill in a form to record daily dental sensitivity after bleaching. Patients were instructed in detail on how to perform this procedure. These forms returned to the investigator at every clinical appointment.

For the 4-point numeric rating scale (NRS), we asked the patient to indicate the numeric value of the degree of sensitivity (from 0 to 4) for each of the periods, in which zero means no sensitivity, 1 means mild, 2 means moderate, 3 means considerable, and 4 means severe tooth sensitivity. In addition, the participants were also instructed to record pain intensity using the visual analog scale (VAS). This scale is a 10-centimeter horizontal line with scores of zero and ten at each end, in which 0 means no sensitivity, and 10 means severe tooth sensitivity. The patient was required to mark with a vertical line across the horizontal line of the scale the intensity of the tooth sensitivity. Then, the distance in millimeters from the zero end was measured with the aid of a millimeter ruler.

We merged the daily data from the three weeks of bleaching for statistical purposes. For this purpose, the worst score (NRS scale) and the highest numerical value (VAS scale) from the total period were taken to represent the patient’s sensitivity level throughout the study. If the participant scored zero (no sensitivity) in all time-assessments, this participant was considered to be insensitive to the bleaching protocol. In all other circumstances, the participants were believed to have bleaching-induced tooth sensitivity.

#### Gingival irritation evaluation

Participants were instructed to fill out a form to record the daily GI after bleaching. These forms returned to the researcher at the next appointment (with one-week intervals), during the three weeks of treatment. For the GI questionnaire, the participant was asked to indicate if they felt any discomfort in the gingiva and if there was discomfort, the side should be indicated.

As with tooth sensitivity assessments, when the participants reported no gingival irritation at all three-week bleaching time evaluations, they were considered insensitive to the bleaching protocol. In all other circumstances, participants were considered to have GI induced by bleaching.

## Statistical analysis

**Figure 1:**
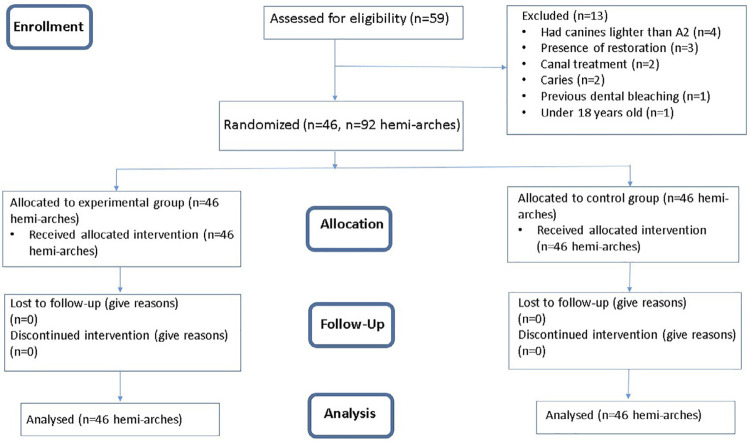
Flow diagram of study design phases including enrollment and allocation criteria

All participants received the intended protocol and had their outcomes measured, meaning that the intention-to-treat protocol and per-protocol analysis resulted in the same findings ( Figure 1 ). The statistician was blinded to the groups.

Two one-sided t-tests for paired samples (TOST-P) were used to test the equivalence of the study groups at the different assessment points (baseline vs. 1-week; baseline vs. 2-week and baseline vs. 3-week and baseline vs. 1-month post-bleaching). Such an approach includes a right-sided test for the lower margin of the equivalence limit and a left-sided test for the upper margin using one-sided 0.025 significance levels. The overall p-value is taken to be the larger of the two p-values from the lower and upper tests. Mean difference, and 95% confidence interval (CI) were calculated between groups at each time assessment. For ΔE, if both treatments differ by more than 3.0 units in either direction, then equivalence does not hold. Although not powered for, we similarly evaluated equivalence for color change in shade guide units (defined as a change in 1.0 shade guide unit for both shade guide scales). A traditional one-way repeated-measures ANOVA was employed for each color change instrument to detect color changes over time.

The risks of TS and GI of both groups were compared using the McNemar’s exact test, which is used to compare the proportion of paired data (α = 0.05) in superiority trials. The odds ratio, as well as the confidence interval (CI) for the effect size also was calculated. The Wilcoxon Signed Rank test compared the dataset of TS intensity obtained with the NRS scale while the paired Student t-test compared the TS intensity from VAS scale (α=0.05).

Correlation coefficients in paired designs are essential to allow more precise sample size calculation for future randomized clinical trials. We calculated the phi correlation coefficient for pairs of binary data of the risk of TS and GI between the two groups. The Spearman and Pearson correlation coefficient was calculated respectively for the NRS scale and VAS scale. We used the Pearson correlation coefficient to calculate the correlation coefficient for the pairs of color change for each instrument for the baseline vs. 1-month post bleaching time.

## Results

We examined a total of 59 participants according to the inclusion and exclusion criteria, but only 46 participants remained for the clinical trial ( Figure 1 ). The baseline color of the participants was 9.8 ± 2.3 in shade guide units measured with the Vita Classical guide. The mean age was 24.2 ± 5.1 years and approximately 60% of them were females. All participants attended the recall visits during the bleaching protocol, and none quit the treatment, as seen in Figure 1 .

### Primary outcome color change

**Figure 2:**
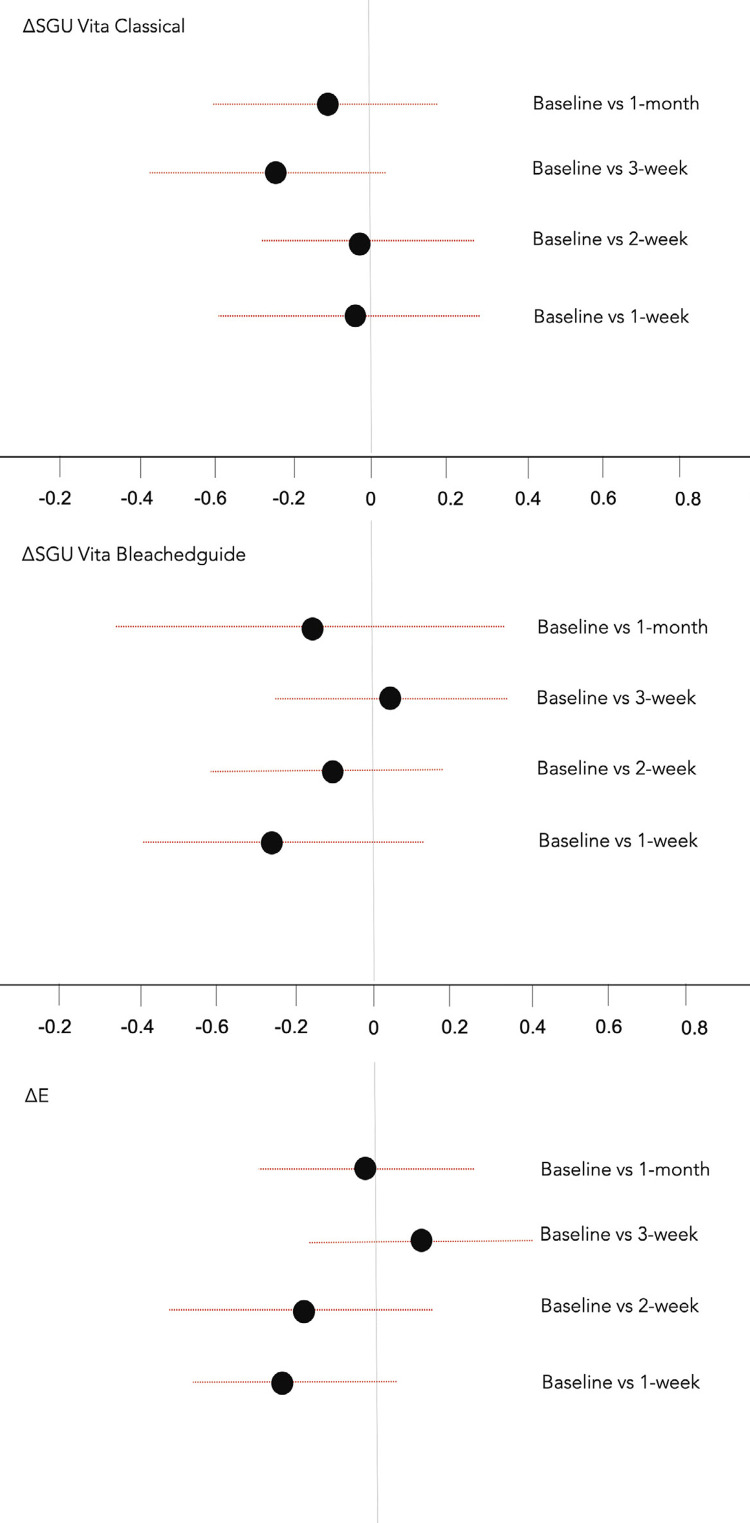
Mean differences of color change measured with different instruments between non-reservoir and reservoir groups at the different assessment times. Horizontal bars indicate two-sided 90% confidence interval (CI) of the mean difference between treatment groups. The zone between the dashed lines indicates the equivalence margin

Table 1 presents the mean differences in color changes for both treatment groups and this can be visualized in Figure 2 . The TOST test demonstrated the equivalence of color change for ∆E, ∆E00, ∆Wi, ∆SGU from Vita Classical scale and ∆SGU from Vita Bleached guide. The two-sided 90% CI of the difference of the means are within the predetermined equivalence margins of -3 and +3 for ∆E, ∆E00 and ∆Wi, and -1 and +1 for ∆SGU.

**Table 1:** Means ± standard deviations of ΔSGU, ΔE, ΔE00 and ∆Wi obtained by the color change instruments at different time assessments and the mean difference (90% confidence interval [CI]) for the pairwise comparison

Color evaluation tool	Time assessments	Groups		Mean difference (90% CI)	Equivalence (p-value*)	Main factor time**
		With reservoirs	Without reservoirs			
Vita Classical	Baseline vs. 1-week	4.7 ± 2.0	4.8 ± 1.8	-0.1 (-0.4 to -0.2)	Yes, p < 0.01	4.8 ± 2.6 ^A^
	Baseline vs. 2-week	7.3 ± 2.0	7.5 ± 1.9	-0.2 (-0.5 to 0.1)	Yes, p < 0.01	7.4 ± 2.3 ^B^
	Baseline vs. 3-week	8.3 ± 2.1	8.3 ± 2.0	-0.0 (-0.3 to 0.3)	Yes, p < 0.01	8.3 ± 2.0 ^C^
	Baseline vs. 1-month	7.9 ± 2.2	7.9 ± 2.0	-0.0 (-0.4 to 0.3)	Yes, p < 0.01	7.9 ± 2.0 ^B,C^
Vita Bleached	Baseline vs. 1-week	5.2 ± 2.3	5.6 ± 2.3	-0.3 (-0.6 to 0.1)	Yes, p < 0.01	5.5 ± 3.7 ^A^
	Baseline vs. 2-week	9.3 ± 2.6	9.6 ± 2.4	-0.1 (-0.4 to 0.2)	Yes, p < 0.01	9.4 ± 3.1 ^B^
	Baseline vs. 3-week	11.6 ± 2.5	11.6 ± 2.3	0.0 (-0.3 to 0.3)	Yes, p < 0.01	11.6 ± 2.4 ^C^
	Baseline vs. 1-month	10.5 ± 2.9	10.7 ± 2.6	-0.2 (-0.7 to 0.3)	Yes, p < 0.01	10.6 ± 2.7 ^D^
ΔE	Baseline vs. 1-week	9.3 ± 4.6	10.1 ± 4.6	-0.8 (-1.5 to 0.1)	Yes, p < 0.01	9.5 ± 4.5 ^A^
	Baseline vs. 2-week	11.8 ± 3.7	12.5 ± 4.1	-0.6 (1.64 to 0.4)	Yes, p < 0.01	12.0 ± 4.3 ^B^
	Baseline vs. 3-week	14.2 ± 3.4	13.9 ± 3.5	0.29 (0.54 to 1.12)	Yes, p < 0.01	14.1 ± 3.4 ^C^
	Baseline vs. 1-month	13.4 ± 3.4	13.5 ± 3.5	-0.12 (-0.93 to 0.69)	Yes, p < 0.01	13.5 ± 3.8 ^C^
ΔE00	Baseline vs. 1-week	5.7 ± 3.1	6.1 ± 3.1	-0.39 (-1.47 to 0.69)	Yes, p < 0.01	5.9 ± 3.1 ^A^
	Baseline vs. 2-week	7.4 ± 2.3	7.7 ± 2.8	-0.31 (-1.21 to 0.59)	Yes, p < 0.01	7.5 ± 2.5 ^B^
	Baseline vs. 3-week	9.0 ± 2.4	8.8 ± 2.3	0.19 (-0.63 to 1.01)	Yes, p < 0.01	8.9 ± 2.3 ^C^
	Baseline vs. 1-month	8.3 ± 2.2	8.4 ± 2.4	-0.09 (-0.9 to 0.72)	Yes, p < 0.01	8.4 ± 2.3 ^C^
∆Wi	Baseline vs. 1-week	13.3 ± 7.2	14.7 ± 6.8	-1.38 (-3.83 to 1.07)	Yes, p < 0.01	14.0 ± 7.0 ^A^
	Baseline vs. 2-week	19.7 ± 6.8	20.1 ± 7.7	-0.34 (-2.87 to 2.19)	Yes, p < 0.01	19.9 ± 7.2 ^B^
	Baseline vs. 3-week	22.9 ± 6.7	22.4 ± 7.1	0.46 (-1.94 to 2.86)	Yes, p < 0.01	22.6 ± 6.9 ^B,C^
	Baseline vs. 1-month	24.1 ± 6.8	22.8 ± 7.3	1.29 (-1.17 to 3.75)	Yes, p < 0.01	23.4 ± 7.0 ^C^

* The p-value reported is the larger of the two p-values from the upper and lower one-sided tests (TOST test); ** One-way repeated measures ANOVA (p < 0.05).

The traditional one-way repeated ANOVA measures detected significant whitening over time ( Table 1 ; p>0.05). After one month, a whitening effect of near eight units in the Vita Classical scale and 11 units in the Vita Bleached guide were observed. Regarding to spectrophotometers measurements, we observed a color change of 13 units for ∆E, 9 units for ΔE00 and 22 units for ΔWi ( Table 1 ).

The Pearson correlation for the baseline vs. 1-month post bleaching in the ∆SGU from Vita Classical was 0.80 (p<0.01); from the Vita Bleached guide it was 0.74 (p<0.01), for ∆E was 0.55 (p<0.01), for ∆E00 was 0.65 (p<0.01) and ∆Wi was 0.83 (p<0.01).

### Secondary outcomes

#### Risk of tooth sensitivity

A total of fifteen patients reported pain in the experimental arch side (absolute risk: 33%, 95% CI 21 to 46%), and from these, 5 patients did not report pain in the control side. Sixteen patients (absolute risk: 35%, 95% CI 23 to 49%) reported pain in the control group, and from these, 6 did not experience pain in the experimental side. In relative terms, the odds ratio for pain was 0.8 (0.2 to 3.0; Table 2 ), and thus it did not reach statistical significance (p=1.0, McNemar’s test). The Spearman correlation coefficient for pairs of binary data was moderate and significant (r=0.47; p<0.01).

**Table 2:** Matched tabulation of the absolute risk of tooth sensitivity for both groups along with the odds ratio and 95% confidence interval

		Without reservoirs			Odds ratio
		Positive	Negative	Total	(95% CI interval)
With reservoirs	Positive	10	5	15	0.8 (0.2 to 3.2)
	Negative	6	25	31	
	Total	16	30	46	

McNemar’s test (p=1.0); Spearman correlation between paired data =0.47; p-value=0.0001.

#### Risk of gingival irritation

A total of 16 patients reported pain in the experimental arch side (absolute risk: 35%, 95% CI 23 to 49%), and from these, 4 patients did not report pain in the control side. Seventeen patients reported pain in the control group (absolute risk: 37%, 95% CI 25 to 51%), and from these, 5 did not experience pain in the experimental side. In relative terms, the odds ratio for pain was 0.8 (0.16 to 3.7; Table 3 ), and thus it did not reach statistical significance (p = 1.0, McNemar’s test). The Spearman correlation coefficient for pairs of binary data was moderate and significant (r=0.57; p<0.01).

**Table 3:** Matched tabulation* of the absolute risk of gingival irritation for both groups along with the odds ratio and 95% confidence interval

		Without reservoirs			Odds ratio
		Positive	Negative	Total	(95% CI)
With reservoirs	Positive	12	4	16	0.8 (0.16 to 3.7)
	Negative	5	30	30	
	Total	17	29	46	

* McNemar’s test (p=1.0). Spearman phi correlation between paired data =0.47; p=0.0001.

#### Intensity of tooth sensitivity

The statistical analysis did not show any significant difference in the TS intensity between groups in any of the pain scales (p=0.64 for NRS scale, and p=0.23; for VAS scale; Table 6). The mean difference of pain intensity in VAS scale was on average - 0.2 units, a difference far from clinically important. Pain was positively correlated in both groups ( Table 4 ). Correlation was moderate and significant for both pain scales. For NRS scale, the Spearman correlation was 0.52 (p<0.01) and for VAS scale, the Pearson correlation was 0.69 (p<0.01).

**Table 4:** Intensity of tooth sensitivity for both groups, mean difference of the paired VAS means and mean difference along with 95% confidence interval [CI].

Pain scales	With reservoirs	Without reservoirs	Mean difference (95% CI)	p-value
NRS 0-4	1 (0 – 1.25)	1 (0 – 1)	--	0.64*
VAS 0-10	1.5 ± 1.7	1.7 ± 2.0	- 0.2 (-1.0 to 0.6)	0.23**

For NRS scale, the values reported are medians and the interquartile range. For VAS scale, the values are reported in means and standard deviations. *Wilcoxon Signed Rank test; **Paired t-test.

## Discussion

The use of reservoirs in the bleaching trays was initially seen as positive, since higher accumulation of material could provide the patient with greater treatment efficacy. ^11^ After the emergence of this new technique, some clinical trials ^11 , 13 , 14^ observed that the efficacy of this treatment was not dependent on reservoirs but rather in the exposure area and gel application time. ^10^ However, although these studies reported these findings, they were considered at unclear risk of bias in a recent systematic review. ^15^ Additionally, these earlier studies had low statistical power. Negative results of low-powered studies may not indicate that one group is different from another, but rather that these results may be due to chance alone.

These earlier studies ^10 , 11 , 13 , 14 , 16 - 18^ also lacked the use of standardized outcomes to report their findings of color change, tooth sensitivity and gingival irritation, which reduced the reliability of the study’s findings. The limitations above of the previous studies on this issue motivated us to conduct this randomized clinical trial.

In the present study, we measured color change by using subjective methods (matching with different shade guide units) along with objective methods (spectrophotometer). It is reported that measurement with a spectrophotometer provides more accurate results than visual shade matching with shade guides ^25 , 26^ as it is less prone to subjective judgments; however, results published in ∆E are less clinically tangible. It worth to mentioning that, in this study the CIEDE2000 system and Whiteness Index for Dentistry were also used. ^23 , 24^ According to Sharma, Wu and Dalal ^27^ (2005) the CIEDE2000 is more compatible with visual color alteration perception and acceptance because the color measurement is adjusted by light, hue, and chroma parameters. The Whiteness Index for Dentistry allowed for more information on whiteness effect, a very important parameter in bleaching studies. ^28^ Although these advantages of CIEDE2000 and Whiteness Index for Dentistry, all color measurements scales showed the same results.

However, it worth to mentioning that the Vita Classical scale and CIELAB 1978 are still the most used parameters to measure color change in clinical bleaching trials. ^29 , 30^ Therefore, their use allows the data to be compared with different clinical trials and could help to improve the scientific evidence. This explains why we have reported color change using several different tools. Also, only a few published studies that evaluated color change using bleaching trays with and without reservoirs included objective tools for color change in their analysis. ^10 , 18^

Significant whitening effects of approximately eight units in the Vita Classical scale and 11 units in the Vita Bleached guide scale, 13 units for ∆E, 9 units for ΔE00 and 22 units for ΔWi were observed in the sides with and without reservoirs. Equivalence was demonstrated in all time-assessments, regardless of the tool used for color change. The results of this study suggest that the efficacy of at-home bleaching is not related to the amount of bleaching material presented at the buccal surface of teeth to be bleached. A similar finding was reported by a randomized controlled trial that showed no difference in efficacy when 10% hydrogen peroxide was delivered in a bleaching strip or customized/prefilled bleaching trays. In the latter, the amount of bleaching product is significantly higher ^31^ than in the bleaching strip. Other factors such as concentration, ^7 , 32^ daily usage time, ^33^ total treatment time ^34^ and degradation kinetics of the product ^35^ may be more important than the amount of bleaching gel on the bleaching tray.

Bleaching-induced TS is directly related to the flow of hydrogen peroxide to the pulp chamber. ^36^ As the presence of reservoirs offers a higher amount of bleaching gel, it is believed that the tooth sensitivity could be aggravated, as well as gingival irritation. ^13^ However, the present study showed that there is no significant difference between groups whitened with and without reservoirs for gingival irritation or tooth sensitivity. Approximately 30% of the patients reported tooth sensitivity with a very low intensity (about 1.6 unit in the VAS scale), and no type of additional desensitization was required.

The similar risk of TS between both groups reinforces the fact that the amount of material placed on the enamel surface does not affect the bleaching outcome. The penetration of the bleaching agent is not driven by the mass (amount) of product placed on the surface but by the diffusion coefficient of the bleaching product itself on the dental substrate. ^10 , 33^ This diffusion coefficient is dependent on the nature of the substance under diffusion and on the area of application. Factors such as viscosity and solution properties (concentration, pH and, temperature) which were not altered between groups can affect diffusion, but not the product mass. ^33 , 34^ Therefore, one can expect that the amount of hydrogen peroxide that achieved the pulp chamber was similar in both sides of the patient’s arches, leading to a similar risk of bleaching-induced TS; this may explain the fact that our findings agree with previous studies, which also found no differences in TS between groups with and without reservoirs. ^10 , 11 , 14 , 16^

For calculation of risk and intensity of TS, we summarized the tooth sensitivity data based on the worst episode of pain in the bleaching period. In the author’s opinion, the experience of considerable pain makes the experience negative for patients, even if it is a single episode or multiple episodes. This provides us with the worst scenario; other ways to report the adverse effect of tooth sensitivity, however, do exist. For instance, in an exploratory analysis we calculated the mean number of days patients experienced tooth sensitivity (reservoir side: 3.9 ± 5.0; non-reservoir side; 4.6 ± 5.9), and also the intensity of TS (VAS scale) by taking the mean of the daily TS during the bleaching period (reservoir: 0.8 ± 1.0; non-reservoir: 0.9 ± 1.0). By using appropriate statistics, we reached up with the conclusion of no significant difference between groups which makes the results of the present investigation robust and not affected by these prior decisions.

Apart from not bringing benefits to the at-home bleaching, designing bleaching trays with reservoirs will require a more significant amount of bleaching material used to whiten teeth, as well as more time for tray fabrication, increasing the costs associated with this procedure. Some companies, such as Ultradent Products still recommend the manufacturing of bleaching trays with reservoirs. This recommendation is not based on the findings of controlled trials but probably on the fact that more material is required to fill in the bleaching tray with reservoirs. While this may be an advantage for the company, the same is not true for for clinicians and patients, who will spend more in bleaching material than one would if bleaching was performed with trays without reservoirs. Unfortunately, clinicians have much more access to the manufacturer’s instruction of bleaching products than access to findings of randomized clinical trials or even laboratory studies, which also found no advantages in the presence of reservoirs. ^37^

Finally, we should mention the limitations of the present study. We have just evaluated one brand of material in this clinical trial. Although this may be seen as a limitation, bleaching agents have very similar composition which contrasts with the majority of the dental materials used in the daily practice. Researchers should conduct further clinical trials using different brand of materials. As the majority of the participants were young in this clinical trial, results should not be generalizable to older populations without care.

## Conclusion

The presence or absence of reservoirs in a bleaching tray did not affect color change, tooth sensitivity, or gingival irritation in a dentist-supervised, at-home bleaching performed with 10% carbamide peroxide gel.
